# Crosstalk between Inflammation and Atherosclerosis in Rheumatoid Arthritis and Systemic Lupus Erythematosus: Is There a Common Basis?

**DOI:** 10.3390/life14060716

**Published:** 2024-05-31

**Authors:** Marta Chiara Sircana, Gian Luca Erre, Floriana Castagna, Roberto Manetti

**Affiliations:** Department of Medical, Surgical and Pharmacology, University of Sassari, 07100 Sassari, Italy; glerre@uniss.it (G.L.E.); floriana.castagna@aouss.it (F.C.)

**Keywords:** autoimmunity, rheumatoid arthritis, systemic lupus erythematosus, atherosclerosis, cardiovascular risk

## Abstract

Cardiovascular disease is the leading cause of morbidity and mortality in patients with rheumatoid arthritis and systemic lupus erythematosus. Traditional cardiovascular risk factors, although present in lupus and rheumatoid arthritis, do not explain such a high burden of early cardiovascular disease in the context of these systemic connective tissue diseases. Over the past few years, our understanding of the pathophysiology of atherosclerosis has changed from it being a lipid-centric to an inflammation-centric process. In this review, we examine the pathogenesis of atherosclerosis in systemic lupus erythematosus and rheumatoid arthritis, the two most common systemic connective tissue diseases, and consider them as emblematic models of the effect of chronic inflammation on the human body. We explore the roles of the inflammasome, cells of the innate and acquired immune system, neutrophils, macrophages, lymphocytes, chemokines and soluble pro-inflammatory cytokines in rheumatoid arthritis and systemic lupus erythematosus, and the roles of certain autoantigens and autoantibodies, such as oxidized low-density lipoprotein and beta2-glycoprotein, which may play a pathogenetic role in atherosclerosis progression.

## 1. Introduction

Inflammation is the body’s natural response to harmful stimuli and is an essential component of the immune response that enables host survival against infection. However, when this process is excessive and dysregulated, it damages homeostasis and impairs organ function [1,2]. In the cardiovascular system, the first impact of inflammation is the promotion of atherosclerosis [3,4].

Autoimmune diseases represent a rich field of research, but their cardiovascular risk is still insufficiently understood. The two most common autoimmune connective tissue diseases are rheumatoid arthritis (RA) and systemic lupus erythematosus (SLE).

SLE is an autoimmune disease characterized by a wide spectrum of manifestations and mainly affects young women [5], and RA is an autoimmune disease that primarily affects the synovial joints of female patients, characterized by symmetrical polyarthritis with potential systemic involvement [6].

The pathogenesis of these two diseases is not yet fully clarified, but it is believed to be due to a complex interplay between genetic and environmental factors, including microbial triggers, in the context of a dysregulated immune system with excessive inflammatory load due to inflammasome activation and pro-inflammatory cytokines.

Subclinical atherosclerosis is common in patients with RA, independently of traditional cardiovascular risk factors [7] and its progression has been observed 3–5-fold more in patients with RA than in individuals without autoimmune diseases [8]. A similar trend has also been observed in patients with SLE; pediatric populations have shown endothelial dysfunction [9], higher rates of atherosclerotic plaque have been found in SLE patients compared with controls in most studies [10,11], and there is an accelerated progression rate of atherosclerosis (ATS) in SLE, with an average 10% annual increase in atherosclerosis compared to <5% in the general population [12]. The influence of traditional cardiovascular risk factors on carotid plaque formation may be similar in patients with RA and patients with SLE [13].

As regards cardiovascular (CV) morbidity and mortality in RA, the risk of cardiovascular events (i.e., myocardial infraction and stroke) is estimated to be 1.5 times higher than in healthy controls [14]. Non-fatal cardiovascular events are also increased in SLE patients [10].

Patients with SLE have a risk of atherosclerotic cardiovascular disease (CVD) almost twice as high as the healthy general population when compared by age and gender, thus comparable to the risk of patients with type 1 diabetes mellitus [15]. Furthermore, according to a the Medicaid Analytic eXtract review, SLE patients had a 27% higher risk of non-fatal CVD events compared with age- and gender-matched diabetic patients [16].

CVD is the first cause of mortality in RA, accounting for 40–50% of deaths [17,18], and in lupus patients, CVD is a leading cause of death after kidney disease and infections [19].

Compared to the general population, the risk of death in lupus patients has been found to be two to five times higher [20,21]. Male gender, black race and renal manifestation were associated with higher mortality [22,23].

Mortality in RA is 1.5–1.6-fold higher than in the general population [24].

Accordingly, cardiovascular risk scores based on traditional risk factors appear to perform sub-optimally in these patient groups. Clinical cardiovascular risk scores such as the Framingham score (FRS), System for Cardiac Operative Risk Evaluation (SCORE) and QRSEARCH underestimate cardiovascular risk in patients with lupus and RA and evidence of atherosclerosis [25].

To date, only the Reynolds Risk Score (RRS) [26,27] includes the measurement of a sensitive index of inflammation, C-reactive protein (CRP) [28] and only QRISK3 [29] includes RA and SLE as independent CV risk factors. Therefore, the prediction of future cardiovascular events in RA and SLE should take in account disease-specific risk factors that can accumulate accelerating atherosclerotic plaque evolution [30]. The Expanded Cardiovascular Risk Prediction Score for RA (ERS-RA) predicts the 10-year risk of cardiovascular events in the RA population based on traditional and unconventional RA-specific (disease duration, disease activity, functional capacity and glucocorticoid use) risk factors [31]. In a large cohort of Italian RA patients without previous cardiovascular events, a 20 mg/L increase in CRP concentrations was associated with a 1% increase in the 10-year risk of CV events calculated with the ERS-RA score [32,33].

In light of the lack of adequate precise data, a multiplication factor of 1.5 at the beginning of RA has been retained in the recently updated European League Against Rheumatism (EULAR) recommendations, regardless of any disease characteristic criteria [34], but for SLE and antiphospholipid syndrome, the EULAR recommendations do not endorse the use of a specific CV risk assessment tool due to limitations and discrepancies in the evidence [35,36].

## 2. Pathophysiology of Atherosclerosis

The events leading to the formation of atherosclerotic plaque can be summarized by three basic stages:

– Injury to the endothelium;
– An interaction between the arterial wall, lipoproteins and platelets;
– Smooth muscle cell proliferation.

Finally, the influx and activation of inflammatory cells perpetuates the vicious cycle of inflammation, which can lead to atheroma lesion complications [37].

The first step in atherogenesis is endothelial damage. An endothelial lesion may be a consequence of functional damage (such as that induced by hypertension, diabetes, hyperhomocysteinemia, lipoprotein (A) or endothelial dysfunction, caused by an imbalance between vasoconstrictive (endothelin) and vasodilating (nitric oxide, NO) substances in favor of the former, and the formation of ROS in the damaged endothelial cells.

As a result of alterations in shear stress and the tangential force exerted by the blood on the endothelium, the endothelium changes from a physiological quiescent phenotype (stimulated by laminar flow) to a dysfunctional, proinflammatory phenotype (stimulated by retrograde flow and low mean shear stress) incapable of adequate NO synthesis.

The reduced bioavailability of NO triggers the production of pro-inflammatory cytokines including IL-1β, IL-6 and TNF-α and reduces the ability of the endothelium itself to produce antithrombotic agents such as prostacyclin (antiplatelet).

Endothelial dysfunction favors the accumulation of low-density lipoproteins (LDL) in the vascular wall. This phenomenon is induced not only by increased permeability of the damaged endothelium, but also by the binding of ox-LDLs to the intimal extracellular matrix.

Subsequently, the LDL accumulated in the arterial intima is modified (aggregation, oxidation and/or glycosylation) and converted into pro-inflammatory molecules (ox-LDL), which, in turn, contribute to endothelial damage, leading to the expression of vascular cell adhesion molecule-1 (VCAM-1) and intercellular adhesion molecule-1 (ICAM-1) and the release of chemokines necessary for the recruitment of monocytes (CCL2 and CCL5) and T lymphocytes from the bloodstream to the intima.

The hemodynamic stress produced by the hydrostatic pressure in the vessel and the transmural pressure (Laplace’s law) acts on the entire vessel wall and induces adaptive intimal thickening: hyperplasia of the normal constituents of the arterial wall, the connective matrix and smooth muscle cells. Hemodynamically, excessive circumferential pressure is countered by a reduction in vessel diameter and an increase in wall thickness and resistance.

Subsequently, the continuous influx of inflammatory cells sets the stage for atheromatous lesion complications to occur.

Circulating monocytes differentiate into macrophages under the influence of local stimuli and increase the expression of scavenger receptors (SRs) and Toll-like receptors (TLRs). Macrophages take up oxidized low-density lipoproteins (LDL) in the intima via SRs, phagocytose them and transform into foam cells (through the intracellular accumulation of cholesteryl esters).

Macroscopically, fatty streaks appear as flat, sharp-edged, reversible lesions. They may regress in the absence of cardiovascular risk factors, or they may progress and become increasingly infiltrated with lipids and foam cells.

Inflammatory cytokines and growth factors, mainly derived from macrophages, and platelet-derived growth factor (PDGF), produced by the endothelium, cause smooth-muscle fibrocells and fibroblasts to migrate from the media into the intima, and the lipid core enlarges.

Type 1 T helper cells (Th1) further activate monocytes via IFN-γ, while regulatory T cells reduce inflammation and support extracellular matrix (ECM) deposition via IL-10 and TGFβ.

The progression of the inflammatory process leads to the further recruitment and activation of neutrophils, monocytes, and T and B lymphocytes towards the lesion, which macroscopically takes the appearance of a plaque: a raised and circumscribed lesion covered by a fibrous cap.

At the center of atherosclerotic plaques is the necrotic core, which is formed by the death of macrophages and impaired efferocytosis, a process that removes apoptotic cells.

Calcification is another hallmark of advanced atherosclerosis.

The fibrous cap separates the thrombogenic necrotic core from circulating clotting factors and platelets, and its thickness correlates with plaque vulnerability. The plaque can be stabilized by fibrous cap thickening or stabilized by destruction of the lipid cap by macrophage metalloproteases and local necrosis processes.

The latter can lead to atherosclerotic plaque complications, including rupture, thrombosis, or bleeding.

Inflammatory cytokines such as IL-1β, IL-6 and TNF-α produced by macrophages (as well as the loss of the anticoagulant function of the injured endothelium) stimulate the expression of tissue plasminogen activator (PAI-1) and von Willebrand factor (vWF), leading to a procoagulant state [38].

Platelets, which tend to stick to the intima, contribute to the growth of the atheromatous plaque. If the excessive inflammation of the necrotic core has ulcerated the fibrous cap, the subendothelial space is exposed, as is the thrombogenic necrotic core, leading to thrombus formation.

Alternatively, hemorrhaging of the plaque can occur. The development of microvascular plexuses connected with the vasa vasorum of the artery is responsible for the growth of the atherosclerotic lesion. Neovessels provide a large area for leukocyte trafficking; these fragile vessels can lead to focal hemorrhages that stimulate fibrosis and thrombus formation.

Acute myocardial infarction (MI) and ischemic stroke are often clinical consequences of occlusive thrombosis on a ruptured plaque.

In addition, emerging evidence points to a role of viral and bacterial triggers in the pathogenesis of atherosclerosis [39], triggers that are also implicated in the pathogenesis of autoimmune diseases [40], hepatitis viruses, periodontal bacteria, LPS, etc., due to molecular mimicry phenomena. Further research has revealed the presence of autoantibodies that can enhance the inflammatory process, such as antibodies against glucose-regulated protein 78 (GRP78) and those against heat shock proteins 60 and 90 (HSP60, HSP90) [39].

Heat shock proteins are chaperones that act in the mitochondrion, where they regulate apoptotic or anti-apoptotic cell functions, including inflammatory responses, and migrate to the cell surface, where they increase the expression of leukocyte adhesion molecules. Once on the cell surface, they can be targeted by autoantibodies present in the serum of patients with infectious diseases. Such autoantibodies are present in significantly higher titers in patients with coronary artery disease than in controls [41].

GP78 acts as an autoantigen expressed on endothelial cells and can be targeted by anti-GRP78 antibodies. Such autoantibodies can enhance the inflammatory process by activating the NFKB pathway in mouse models, thereby reducing the stability of atherosclerotic plaques and accelerating their growth [42]. And they are present in higher titers in patients with cardiovascular disease than in controls.

This emerging evidence points to the need for further research to address the inflammatory pathogenetic molecular mechanisms at the basis of atherosclerosis and to identify new molecular targets for the therapy of atherosclerosis.

It is reasonable to assume that the modulation of inflammation may affect both the genesis of the plaque (endothelial dysfunction, LDL oxidation) and the complications of atherosclerosis, either by accelerating them, as in autoimmune and inflammatory diseases, or by slowing their progression, as with disease-modifying antirheumatic drugs (DMARDs) and lifestyle.

## 3. Rheumatoid Arthritis

RA is a chronic inflammatory disease that typically affects small to medium-sized joints in a symmetrical, centripetal pattern.

RA affects about 0.5–1% of adults in Europe and North America. Women are about three times more likely to be affected than men, with a peak age of onset between 50 and 60 years of age. The primary injury is synovitis, leading to cartilage destruction, bone erosion and, ultimately, loss of joint function. The disease can also have systemic consequences, and uncontrolled RA is associated with a 6–7-year reduction in life expectancy.

Rheumatoid factor (RF) and anti-citrullinated protein antibodies (ACPA) are important in the diagnosis and prognosis of the disease, as the presence of RF and ACPA is associated with a more severe course of the disease.

The pathogenesis of RA is not yet understood. It is thought that in genetically predisposed individuals, an environmental factor can deceive the immune system (molecular mimicry) or modify antigens that should be recognized by the immune system as self; this disrupts immunological tolerance to certain human proteins (such as joint collagen 1), causing the dysregulation of T and B lymphocytes and the subsequent production of inflammatory cytokines.

Although the causative factors of RA are still unknown, many inflammatory chemokines and cytokines are known to play key roles in the development of the disease.

All cells of the immune system (T and B lymphocytes, monocytes, macrophages, neutrophils) and numerous soluble inflammatory mediators are involved in the pathogenesis of RA, particularly proinflammatory cytokines, with the most important including IL-1, IL-6 and TNF-a.

### 3.1. Inflammation in Rheumatoid Arthritis-Associated Atherosclerosis

**Figure 1:**
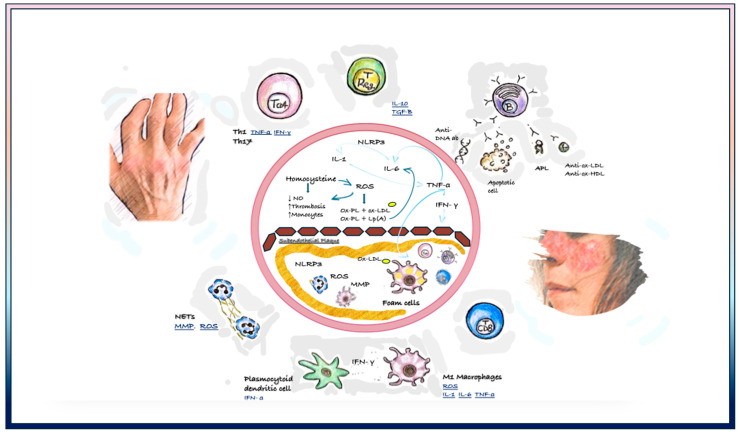
Pro-atherogenic pathways in RA and SLE.

Atherosclerosis and autoimmune connective tissue diseases share common molecular pathways. Each disease has its own unique pro-atherogenic pathway through its own physiopathology, but there are some common pathways (See Figure 1).

Endothelial damage is also the first step in vascular damage in RA patients. Endothelial dysfunction is usually defined as the reduced synthesis of nitric oxide (NO) [43].

Endothelial damage is generally induced by immune complexes and autoantibodies binding to the endothelium, leading to excessive coagulation, vasoconstriction and the development of atherosclerosis.

The following mechanisms have been proposed to cause a reduction in the bioavailability of NO: the direct toxicity of inflammatory cytokines like IL-6 [44], IL-1 [45] and TNF-alpha [46]; an intracellular lack of L-arginine; the reduced bioavailability of the cofactor BH4; the accumulation of the natural inhibitor of eNOS, methylated arginine asymmetric dimethylarginine [47]; and the inactivation of NO due to the excessive production of superoxide anions [48].

Endothelial dysfunction in RA patients has been associated with a reduced number and the dysfunction of endothelial progenitor cells (EPC), characterised by and insufficient migratory response to VEGF stimulation and reduced adherence capacity [49].

Furthermore, chronic inflammation impacts the lipid profile. Oxidized low-density lipoproteins are implicated in endothelial dysfunction in RA and SLE [50]. Oxidized phospholipids that bind to ox-LDL and Lp(A) exert a pro-atherogenic effect [51] and are linked to CVD events [52]. Lp(A) exerts its pro-atherogenic effects via the ox-PL that are bound to it [52] and it promotes macrophage activation [53]. Lp(A) levels are typically elevated in RA and SLE, and IL-6 receptor inhibitors have been demonstrated to reduce them [54], suggesting a potential association between IL-6 and Lp(A).

HDL levels are lower in chronic inflammation (RA and SLE) due to reduced synthesis and increased clearance [55]. Homocysteine reduces NO synthesis [56], stimulates ROS production, induces protein susceptibility to oxidation (thus, they can become new autoantigens) [57] and enhances monocyte proliferation. Homocysteine plays a role in RA joint damage as well as atherosclerosis, thrombosis, and stroke [58].

In RA, pro-inflammatory cytokines, including TNF, IL-1β and IL-6; chemokines; and immune complexes (e.g., with citrullinated proteins) are released into the circulation from the synovial tissue, the bone marrow of inflamed joints or associated lymph nodes. They exert systemic effects leading to cardiovascular disease, including atherosclerosis, insulin resistance, visceral obesity and metabolic syndrome.

The degree of systemic inflammation is strongly associated with the progression of atherosclerosis and the risk of cardiovascular disease (CVD).

Patients with RA are at increased risk of developing CVD, including myocardial infarction (MI), cerebrovascular disease, peripheral arterial disease and congestive heart failure (CHF), with a 48% increased risk of incident CVD, a 68% increased risk of MI, a 41% increased risk of a CV event and an 87% increased risk of CHF (but only in women) discovered compared with the general population in a meta-analysis.

A growing body of literature supports the beneficial role in CVD prevention in reducing the inflammatory burden in patients with inflammatory joint disease with the use of synthetic and biologic DMARDs [59]. In fact, low disease activity appears to slow or ameliorate atherosclerosis regardless of the antirheumatic treatment used (Arida et al., 2017) [60].

### 3.2. Role of Macrophages and the IL-1, IL-6, TNF-Alpha and JAK Pathways in Atherosclerosis and Rheumatoid Arthritis

Macrophages are a critical component of the innate immune system and are specifically part of the mononuclear phagocytic system. When they invade tissues, they differentiate from monocytes.

Macrophages are one of the most abundant innate immune cell populations in RA, and their numbers correlate significantly with disease severity. Synovial tissue macrophages play a major role in the production of inflammatory cytokines responsible for local systemic damage in RA, but also in tissue repair. Their ability to adopt different polarization states explains the discrepancy in their roles.

In particular, two opposing macrophage phenotypes are recognized, M1 and M2, one pro-inflammatory and the other involved in tissue repair. M1 cells are pro-inflammatory, microbicidal, cytotoxic and anti-tumor, while M2 cells are anti-inflammatory and immunoregulatory but can promote tumor progression. These phenotypes can be measured in the patient’s blood and synovial tissue. In patients in drug-induced remission, the pro-inflammatory phenotype is replaced by the anti-inflammatory phenotype, which induces the repair of damaged synovial tissue.

It is important to note that M1 and M2 are an oversimplification; they represent opposite ends of a spectrum of phenotypes and show considerable plasticity. Mantovani et al. demonstrated the plasticity of polarized macrophages by highlighting differences in the expression of receptors, effector function and the production of cytokines and chemokines.

In addition, M1 macrophages may reduce the inflammatory response (NO production) and M2 may exert antimicrobial functions.

Three major stimuli induce the M1 phenotype: IFN-γ, pathogens and granulocyte–macrophage colony-stimulating factor (GM-CSF). IFN-γ is the primary M1 stimulus produced by Th1 and NK lymphocytes and macrophages themselves. TNF-a produced by APC cells also induces M1 polarization. Superantigens such as LPS (lipopolysaccharide) are derived from Gram- bacteria and LTA (lipoteichoic acid) is produced by Gram+ bacteria [61].

Macrophage polarization is associated with a shift in the metabolic program.

M1 and M2 macrophages show a drastic shift in amino acid, glucose, lipid and iron metabolism [62].

During articular inflammation, synovial “pannus” formation in the presence of a hypoxic inflammatory environment dramatically increases glycolytic activity in macrophages, which are polarized towards an M1 phenotype. TLR4 activates aerobic glycolysis to provide sufficient bioenergetic resources for cell maturation. In inflamed joints, oxygen levels drop rapidly, while hypoxia factor 1α (HIF-1α) [63] and ROS production increase, followed by the activation of pro-inflammatory genes (IL-1β and IL-6), promoting massive oxidative tissue damage [64].

Ornithine [65] and nitric oxide (NO) [64] are among the most characteristic molecules guiding the polarization of macrophages towards the M1 and M2 active states, respectively.

Proinflammatory macrophages have increased antigen-presenting capacity, and produce more inflammatory cytokines (IL-1, IL-6, TNFα) and more reactive nitrogen and oxygen intermediates. In addition, their release of IL-12 is increased, promoting the Th1 polarization of CD4+ cells [66]

M1-M2 imbalance may play a role in RA and its comorbidities, and the continuum of phenotypes may be related to the different phases of the disease, i.e., flares and remissions [67].

Due to their excessive activation and proliferation and increased anti-apoptosis capacity, the proportion of M1 macrophages is higher than that of M2 macrophages in RA.

On the other hand, M2 macrophages alleviate inflammation through the production of anti-inflammatory cytokines, including IL-10 and TGF-β, tissue homeostasis and repair, and the activation of T cell regulatory functions.

Historically, AR therapies have targeted inflammatory substances produced by pro-inflammatory macrophages. In recent years, research has also been conducted in the field of drug delivery, such as bioactive nanoparticles and macrophage-derived macrovesicle-coated nanoparticles, to overcome the side effects and limitations of traditional systemic drugs. However, no specific macrophage-targeted treatments are currently in clinical use [68].

The inflammatory immune response of macrophages is also one crucial factor contributing to atherosclerotic plaque formation. A reduction in the macrophage inflammatory response has been associated with anti-atherogenic properties. Many potential macrophage-based treatments for cardiovascular disease target macrophage-produced proinflammatory cytokines [69].

Around 40 years ago, the ‘inflammatory theory of atherosclerosis’ was postulated. It suggests that the dysregulated production of specific cytokines by M1 macrophages, such as tumor necrosis factor-alpha (TNF-α), IL-6 and IL-12, promotes excessive inflammation in the arterial wall.

To clarify the above, the role of macrophage-derived substances in the pathogenesis of atherosclerosis and RA will be briefly reviewed.

In atherosclerosis, IL-1 enhances the production of ICAM-1 and VCAM-1, as well as the production of chemokines such as MCP1, on endothelial cells. This promotes the influx of lymphocytes through the vessels to the site of inflammation, creating a positive feedback loop. IL-1 increases its own expression and induces the production of IL-6 and metalloproteases.

In RA, IL-1 promotes inflammation and osteoporosis, with its function overlapping with that of TNF-α [70]; additionally, it stimulates RANKL, which is an activator of osteoclasts, and inhibits osteoblasts.

It activates matrix metalloproteases (MMPs) and recruits monocytes, macrophages and lymphocytes, which lead to the formation of synovial cloth.

However, targeting IL-1 has not yielded similar clinically effective results in RA to those seen with TNF-α and IL-6 inhibitors. Anti-IL-1 show better results in cardiovascular protection. The CANTOS study (Canakinumab Anti-Inflammatory Thrombosis Outcomes Study) provides compelling evidence that inhibiting the IL-1β/IL-6 signaling cascade significantly reduces cardiovascular risk, regardless of the reduction in circulating lipid levels. The study aimed to evaluate the reduction in major cardiovascular events, such as non-fatal infarction, non-fatal stroke and cardiovascular death, in patients who had previously experienced myocardial infarction and had plasma CRP levels above 2mg/L, and in patients with a normal lipid profile. However, this benefit is offset by an increased risk of serious infections [71].

IL-6 is one of the most abundant cytokines in the synovium in RA. IL-6 exerts several effects: it promotes vascularization and VEGF-mediated vasopermeabilization, it promotes the destruction of cartilage and bone through the recruitment and formation of osteoclasts, and it promotes the differentiation of B lymphocytes producing RF and ACPA, as well as T lymphocytes.

IL-6 may be associated with an increased risk of cardiovascular disease in RA due to its role in systemic and local inflammation. The MEASURE trial evaluated the effect of tocilizumab on biomarkers of cardiovascular disease. Although the drug may increase LDL levels, it also increases HDL and reduces ox-LDL, A-lipoprotein, phospholipase A2 and amyloid A2.

Methotrexate (MTX), a drug with pleiotropic effects on inflammatory cells and cytokine pathways, has been used as a first-line treatment for diseases characterized by systemic inflammation, including RA, since 1948. Despite the advent of new therapies, MTX remains a cornerstone in the treatment of RA due to its efficacy and tolerability.

At low doses, MTX modulates the inflammatory response by inhibiting aminoimidazole-4-carboxamide ribonucleotide transformylase, resulting in increased adenosine levels. This leads to the lower production of pro-inflammatory cytokines (IL-12, IL-6, TNF-α) and an increase in the production of anti-inflammatory cytokines (IL-10 and receptor antagonist for IL-1). MTX also activates macrophages and the T helper type 1 response; additionally, MTX induces the apoptosis of transformed T cells and reduces the production of metalloproteases [72].

A meta-analysis of 15 studies demonstrated a significant reduction in the risk of myocardial infarction in patients with RA who were treated with MTX [73]. Additionally, several studies have shown that methotrexate plays a role in reducing blood pressure [74].

Still, there is no universal consensus among studies on the above results. The CIRT study recruited patients with a history of acute coronary syndrome and diabetes mellitus, metabolic syndrome or both. They were then randomly assigned to receive either a placebo or low-dose MTX. The primary endpoint was the first occurrence of major adverse cardiovascular events, which was defined as a composite of non-fatal myocardial infarction, non-fatal stroke or death from cardiovascular causes; during the follow-up period, MTX did not significantly reduce the levels of IL-1β, IL-6 or hs-CRP compared to the placebo, and it did not reduce the risk of the primary endpoint [75].

In conclusion, based on systematic reviews and meta-analyses of observational studies, MTX may have the potential to be considered for cardiovascular risk management in old RA patients [76].

TNF is a pleiotropic cytokine that acts on most cells via TNF receptor 1 (TNFR1) with pro-inflammatory action. Additionally, TNF modulates the immune response by acting on certain cell types via TNF receptor 2 (TNFR2), including subsets of T and B lymphocytes, fibroblasts and endothelial cells.

TNF is a well-known cytokine in RA and has been extensively studied therapeutically.

In the synovium of patients with RA, TNF induces the proliferation of fibroblast-like synoviocytes, stimulates the production of collagenase by synoviocytes and promotes bone resorption by osteoclasts.

TNF inhibition reduces acute-phase proteins and IL-6 levels, inhibits leukocyte migration, prevents endothelium activation and enhances the function of regulatory T lymphocytes in suppressing the inflammatory reaction.

Several observational studies have shown that TNF inhibitors used in RA therapy reduce the incidence of cardiovascular events. The role of TNF in atherosclerotic disease in the general population is complex [77]. TNF has been demonstrated in the microenvironment of atherosclerotic plaque in humans. It acts on the endothelium by reducing NO production, promoting ROS production and increasing endothelial permeability [78].

TNF-alpha is induced in myocardial tissue by stressful events such as myocardial infarction [79].

However, inhibiting TNF globally may disrupt the balance between adverse and protective signaling pathways in patients with heart failure. Therefore, TNF inhibitors should be avoided in patients with systolic heart failure.

The JAK-STAT pathway is consistently elevated in patients with RA. The inhibition of this pathway intercepts the signaling of different cytokines, including IL-6 and TNF [80].

The JAK-STAT pathway also plays a crucial role in atherosclerosis through its action on IL-6 and TNF. Unlike anti-IL-1, IL-6 and TNF, no large-scale studies have assessed the role of JAK inhibitors in preventing cardiovascular disease in the general population. However, studies on JAK inhibitors have associated them with an increased risk of cardiovascular events among elderly patients with cardiovascular risk factors [81].

### 3.3. The Role of the Inflammasome in RA and Atherosclerosis

Innate immunity relies heavily on pattern recognition. A wide variety of pathogens can be recognized by a small number of pattern recognition receptors (PRRs). The activation of PRRs triggers a rapid inflammatory response, resulting in the production of several inflammatory cytokines, including interleukin (IL)-1β, IL-6 and IL-18 [82].

Inflammasomes are intracellular sensors of harmful agents that elicit an inflammatory response. They are intracellular multi-protein complexes that assemble in response to pathogen-associated molecular patterns (PAMPS) that enter the cell, or danger-associated molecular patterns (DAMPS) that damage cells or tissues.

Inflammasomes assemble in the cytosol of innate immune cells (macrophages, dendritic cells, neutrophils) and endothelial cells, activating caspase-1, which, in turn, activates IL-1β and IL-18, triggering a systemic inflammatory response; caspase-1 induces inflammatory cell death, also known as pyroptosis [82].

Inflammasome Nod-Like Receptor Protein 3 (NLRP3) is an important component of innate immunity, known to be involved in the pathogenesis of atherosclerosis, metabolic syndrome, diabetes, obesity, and inflammatory and autoimmune diseases. The NLRP3 inflammasome is becoming increasingly important in the pathogenesis of RA and its comorbidities, such as atherosclerotic cardiovascular disease, pulmonary interstitial disease and osteoporosis [83].

Some studies postulate that dysfunctional mitochondria (like the ones in autoimmune diseases such as RA and SLE), which release ROS and mitochondrial DNA, may contribute to the activation of the NLRP3 inflammasome [84].

Even though the inflammasome seems to be an optimal target to treat autoimmune diseases and reduce associated CV, drugs that target the NLRP3 inflammatory pathway can have serious adverse effects, such as an increased risk of infection (anakinra, canakinumab and tofacitinib) [83].

### 3.4. Neutrophils in RA and Atherosclerosis

Neutrophils are crucial in the innate immune response, acting through phagocytosis, degranulation, and the formation of neutrophil extracellular traps (NETs). In the synovium of RA patients, neutrophils are the most abundant cells, particularly in the early phases of the disease. They phagocytose immune complexes within the synovia and are responsible for the production of multiple inflammatory cytokines and enzymes that can damage tissue, as well as amplify processes related to adaptive immunity.

Apart from patients in glucocorticoid therapy, an increased neutrophil count in patients with RA is positively correlated with disease activity. Also, the neutrophil-to-lymphocyte ratio seems positively correlated with inflammatory status and RA disease activity [85].

Research on RA patients has primarily focused on the involvement of NETs in protein citrullination. Additionally, increased levels of NETs have been found in the serum of RA patients, which correlate with the levels of systemic inflammatory markers and ACPA.

Some citrullinated autoantigens are present within NETs, and it is possible that at least some of these are released by neutrophils upon death, as these cells are rich in citrullinated proteins themselves [86,87].

NETs also play a role in atherosclerosis and atherothrombosis [88,89,90], as well as in vascular inflammation and coronary atherosclerosis, in SLE [91].

Notably, neutrophils in RA patients produce a high quantity of reactive oxygen species, distinguishing them from those of healthy individuals.

Based on these findings, it is possible to distinguish between different neutrophil phenotypes, one with physiological characteristics and others that display a pro-inflammatory phenotype. This subgroup includes altered neutrophils found in RA, which exhibit delayed apoptosis (due to NfKB signaling), the increased production of ROS and NET formation. In addition, in order to become resident in the joint and to promote inflammation, these RA pro-inflammatory neutrophils down-regulate adhesion molecules and increase the production of chemokines, which attract and activate both innate and adaptive immune cells [87]. In particular, these resident neutrophils attract and activate T lymphocytes, NK cells, monocytes, macrophages and dendritic cells in the synovial space [92]. The elevated oxidative stress they generate coupled with their prolonged survival and their ability to activate other immune cells, thus perpetuating inflammation, has systemic consequences, contributing to endothelial dysfunction and complications of cardiovascular disease.

Several studies have attempted to investigate the effects of various therapeutic agents on neutrophils, given their considerable contribution to the pathophysiology of RA. Among the new therapeutic targets, IL-33, PADI4 and IL-28A have been considered to have some potential; however, the effects of these drugs on cardiovascular disease have not been elucidated.

With regard to DMARDs, which act on pro-inflammatory neutrophils, well-known options include MTX, leflunomide and biologic DMARDs such as tocilizumab (anti IL-6), tofacitinib (JAK inhibitor), rituximab (anti CD20) and anti- TNF-α agents.

Concerning the specific mechanisms of action of these drugs on neutrophils, we will only mention MTX, the cornerstone therapy of RA, as it appears to have the broadest impact on neutrophils in RA. There is increasing evidence that MTX exerts its anti-inflammatory effect by up-regulating adenosine receptors on neutrophils, leading to an increase in adenosine levels. This results in a decrease in cytokine production, particularly TNF-α and IL-1β, the suppression of NF-kB activation, and a reduction in leukocyte accumulation at inflamed sites [93,94].

Low-dose MTX and all other therapeutic agents, except for JAK inhibitors, have a protective effect on both RA and cardiovascular risk by reducing systemic inflammation and intercepting common inflammatory mechanisms involved in atherosclerosis [95].

### 3.5. Role of B and T Lymphocytes in the Pathogenesis of RA and Atherosclerosis

B lymphocytes play a pathogenetic role in RA. Rituximab, a chimeric monoclonal anti-CD20 antibody, causes the rapid depletion of B cells. This therapy is recommended as a third line, after DMARDs and anti TNF inhibitors. Studies have shown that B cell depletion in RA patients leads to improved endothelial function on the background disease activity. However, rituximab may mildly increase lipid levels [96]. Some B cells have atheroprotective functions, including the production of atheroprotective antibodies, so the potential beneficial effect of rituximab on cardiovascular disease needs to be assessed with caution [97].

Among antibodies implicated in the pathogenesis of atherosclerosis in RA, anti-ox-LDL antibodies [98], which cross-react with antiphospholipids and the anti-ox-LDL/β2GPI complex, are all increased both in SLE and RA [99]. This evidence offered a first explanation for the increased cardiovascular risk in autoimmunity [100].

Rheumatoid factor (RF) and antinuclear antibody (ANA) positivity are predictors of CV events and mortality in both those with and without rheumatic disease [100]. However, no significant evidence has been found for increased cardiovascular risk due to CCP positivity. These findings support the role of immune dysregulation in the etiology of CV disease.

When autoantibodies are present, RA patients have accelerated atherosclerosis compared to RA patients who do not have these antibodies. Similarly, autoantibodies in SLE increase atherosclerosis.

The importance of adaptive immunity has been demonstrated in an experimental model of apolipoprotein E-deficient mice. T and B cell deficiency reduce the atherosclerotic burden by 40% to 80% in both the early and late phases of atherosclerotic disease [101].

The T lymphocytes that infiltrate atherosclerotic plaques are mainly T helper (CD4+).

These lymphocytes produce IFN- γ and activate macrophages. IFN- γ reduces collagen synthesis and makes the fibrous cap more vulnerable. Regulatory T lymphocytes exert a protective effect [102].

As mentioned above, there are similarities between the immune cells that are activated in RA and those in atherosclerosis. T helper (Th)1 cells have a pathogenic role in RA joints [103].

Subsequent studies have also revealed the presence of CD4 and CD8 cytotoxic T cells in RA joints. The delayed (type IV) hypersensitivity reaction is the prototype of T cell-mediated inflammation. In this reaction, macrophages that present the target antigen are activated by antigen-specific Th 1 cells that produce interferon IFN-γ.

Before the onset of RA, synovial CD4 T cells express IL-2, TNF-α, IFN-γ, IL-17α, IL-22, IL-4 and GM-CSF.

In RA joints, Tph cells and PD-1high CD4 T cells exhibit antibody-stimulating and proinflammatory functions. They can produce TNF-α, IFN-γ and GM-CSF, as well as IL-21 and CXCL13 [104].

Th17 may only have a marginal role in RA, and anti-IL-17 therapies in RA have been tested in clinical trials with an inadequate response in this disease [105,106]. There was no significant increase in Th17 cells in the peripheral blood (PB) of RA patients compared to healthy controls, and no correlation was found between the frequency of Th17 cells and RA disease activity [107]. Notably, the frequency of Th17 cells in the joint was lower than that of Th1 cells, which contrasts with mouse models of RA [108].

T cell infiltration is present throughout all stages of atherosclerosis. CD4+ T cells become activated by antigens and differentiate into helper T1 (Th1), Th2 and Th17 cells and regulatory T cells (Tregs).

Within the atherosclerotic plaque, T helper cells tend to have a T helper 1 (TH1) or TH17 profile. These cells release pro-inflammatory cytokines that enhance plaque infiltration and inflammation, leading to vulnerable plaques [109]. However, while some studies have reported regulatory and protective roles in selective disease settings, the roles of IL-17 and T helper-17 cells in disease development and plaque stability in atherosclerosis remain unclear and have led to conflicting results [110].

In fact, IL-17 secreted by Th17 cells can promote atherosclerosis, but few studies have reported that IL-17 can also stabilize atherosclerotic plaques. Tregs play a protective role in atherosclerosis, and the Th17/Treg imbalance also plays an important role in atherosclerosis [111].

CD8 T cells have been largely ignored in RA research, mainly because, unlike CD4 T cells, there is little evidence of their involvement. However, CD8 T cells are not only found in the joints of people with RA; their frequency among T cells is actually higher in the joints than in the peripheral blood, and they may adopt an inflammatory phenotype in the synovia of RA patients [112].

Pathogenic studies show that the immune system of RA patients exhibits abnormalities that are comparable to those observed in healthy elderly subjects. Changes in the frequency and phenotype of peripheral T cell subpopulations (a decrease in naive T cells, an increase in memory T cells, loss of the co-stimulatory molecule CD28, contraction of the TCR repertoire), the reduced output of TREC+ thymic recent emigrants and telomere erosion (naive and memory T cells and granulocytes) are hallmarks of the RA immunosenescence fingerprint [113]. Immunosenescence and inflammation, with the progressive loss of protective functions of the immune cells, have a role in atherosclerosis progression as well [114] (Adaptive immunity and atherosclerosis: aging at its crossroads, R. P. Snijckers, soon to be published in Front Immunol. 2024).

RA has long been considered an autoimmune disease mediated by T cells, but it is still difficult to gather sufficient evidence of T cell autoreactivity. T cell autoreactivity in RA joints can be addressed by studying their reactivity to candidate autoantigens. Since the discovery of ACPA, most studies have examined synovial T cell reactivity to citrullinated proteins, including tenacin-C, alpha-enolase and type II collagen. It is unlikely that all joint T cells are self-reactive and pathogenic.

T cell-targeted therapies include abatacept (CTLA-4-Ig) and Alefecept (CD2 antagonist). abatacept is a CTLA4-Ig (cytotoxic T lymphocyte-associated antigen-4-Ig) that acts against RA by inhibiting CD80/86-CD28 interactions (one of the co-stimulatory pathways between antigen-presenting cells and T lymphocytes); it inhibits the formation of immunological synapses.

It is known that the activation of T cells (T CD4+ and T CD8+) requires stimulatory signals after the T cell antigen-specific receptor (TCR) interacts with the antigen–major histocompatibility complex (MHC) of the APC. The T cell is now active. Finally, a third autocrine signal is provided by the production of IL-6. About 48 h after T cell activation, a new transmembrane protein is synthesized, CTLA-4, which is similar to CD28, and binds to the CD80 of the APC but inhibits the immunological synapse, thereby attenuating the hyperactivation of autoreactive T cells. Therefore, abatacept exerts its activity at a more upstream site of action, unlike TNF-α and IL-6 inhibitors, which act directly on inflammatory mediators.

Previous experimental studies have demonstrated the critical role of local CD4 and regulatory T cells in animal models of atherosclerosis [115]. In mouse models, T lymphocytes correlate with the development of accelerated atherosclerosis, and the co-stimulation of CD28-CD80/86 T cells and CTLA4-mediated inhibition also appear to correlate with the severity of atherosclerosis in RA [116].

The ORACLE study, a single-arm observational study, investigated biological interactions between subclinical atherosclerosis in RA patients and immunological disease activity [117]

In addition, abatacept has been compared with TNF-α inhibitors and may improve insulin sensitivity and lipid profiles in RA patients at high risk of accelerated atherosclerosis, similar to diabetic or elderly patients [118].

## 4. Systemic Lupus Erythematosus

Systemic lupus erythematosus (SLE) is a complex autoimmune disease with multisystem involvement, predominantly affecting young women of childbearing age, with a variable clinical course in which latent phases alternate with periods of extreme aggressiveness [119]. Its incidence rates are 6.73 cases per 100,000 people/year for Caucasians and 31.4 cases per 100,000 people/year for Afro-Americans [120]. Women are nine times more frequently affected than men [121,122].

SLE is characterized by profound immune dysregulation, resulting in the formation of autoantibodies, the deposition of immune complexes and damage to multiple organs. The broad spectrum of clinical manifestations can affect various organs, including the joints, skin, nervous system, hemopoietic system, lungs, kidneys, heart and blood vessels. In recent decades, early diagnosis, therapeutic advances and clinical follow-up have reduced the mortality rate of lupus patients, although it remains higher than the general population [123,124].

SLE has a complex etiology involving an interplay of genetic and environmental factors [125]; however, its exact pathogenesis is still unknown [126]. The aberrant activation of autoreactive T and B cells plays a driving role in SLE, with the latter leading to the production of pathogenic autoantibodies and consequent end-organ damage. Antinuclear antibodies (ANA) are present in 95% of SLE patients [127]. Advances in biology have shed light on the role of cells of the innate immune response, APC cells such as plasmacytoid dendritic cells, aberrant neutrophils and macrophages, the reduced clearance of cellular debris and immune complexes, NETs and type I interferons.

### 4.1. Inflammation in Systemic Lupus Erythematous-Associated Atherosclerosis and the Role of Neutrophils, Macrophages, Lymphocytes and Pro-Inflammatory Cytokines

SLE can lead to an increased risk of accelerated atherosclerosis and cardiovascular (CV) events [128]. These include coronary heart disease, cerebrovascular disease and peripheral arterial disease. CV events occur both early and late in the disease course. Younger patients have a much higher risk than their healthy age-matched counterparts.

The prevalence of hypertension in SLE is not entirely explained by the presence of nephritis or glucocorticoid use [129], although a meta-analysis showed that higher doses of corticosteroids are associated with cardiovascular events in SLE [130].

The rates of premature mortality are 2/3 times higher than in the general population [120], and a multicenter study found that approximately 25% of the 10,000 deaths with SLE were caused by CVD [131].

Patients with SLE have a 50 times higher risk of CVD than the general population [132]. In patients with SLE, a higher risk of cardiovascular events was observed in young women, who are generally at low risk of CVD. Children with SLE have an increased rate of progression of carotid intima-media thickness compared with healthy children [133]. A study from the Medicaid database showed that patients with SLE have a 27% increased risk of non-fatal CVD compared to patients with diabetes, which is associated with high cardiovascular risk, and the risk of these two populations is considered equivalent [16].

In both SLE and RA, traditional risk factors contribute to cardiovascular burden alongside disease-specific risk factors, such as disease activity and drug therapy.

Using the QRSEARCH database, a diagnosis of SLE was associated with an increased risk of 115% in women and 55% in men. Although SLE is relatively rare (affecting 0.1% of women and rarely affecting men), the magnitude of the increased risk is high (significantly higher than RA), especially in younger age groups (the hazard ratios were >2 for ages ≤ 45 years) [29].

Regarding traditional CV risk factors, both SLE and RA predispose patients to arterial hypertension [134,135] and insulin resistance [136,137], but only SLE, as opposed to RA, increases the risk of dyslipidemia [138] and hyperhomocysteinemia [139], in particular in subjects with an MTHFR gene polymorphism [140].

The NICE guidelines on lipid modification also highlight the increased risk of CVD associated with SLE; inflammation and an active immune response are thought to be major contributors to excess risk [141].

As regards disease-specific CVD risk factors, in both RA and SLE, we can recognize the chronic inflammatory state, the mean disease activity over time and immune dysregulation. In SLE, we will focus on IFN-I, NETs, autoantibodies, APS syndrome and lupus nephropathy. In addition, some single gene variants have been associated with increased risk for CVD in SLE patients.

Lupus nephritis (LN) is itself a risk factor for CV events, and vascular damage is an often-underestimated cause of progressive renal dysfunction. LN is a severe manifestation of SLE, affecting approximately 40% of patients, and is associated with cardiovascular risk factors through impaired renal function, dyslipidemia and treatment with high-dose glucocorticoids and calcineurin inhibitors [130].

In addition, some genetic variants associated with SLE predispose patients to LN: a single-nucleotide polymorphism of STAT4 has been associated with ischemic cerebrovascular disease and LN, with a severe course [142]. B cell-activating factor (BAFF) mutation is also associated with LN [143].

Lastly, therapies for SLE have different effects on CVD risk; on one hand, glucocorticoids (e.g., the chronic intake of prednisone (even at doses < 7.5 mg/day), azathioprine and cyclophosphamide) can aggravate endothelial dysfunction and plaque formation, increasing CVD risk, and on the other hand, hydroxychloroquine and mycophenolate may possess anti-atherogenic properties.

### 4.2. Endothelial Damage in SLE as a Systemic Effect of Inflammation

Endothelial dysfunction is the starting point for atherosclerosis in SLE.

The vascular damage/protective mechanism in SLE patients is out of balance, caused by a cascade reaction between oxidative stress, pro-inflammatory cytokines, NETs, the activation of B cells and autoantibodies, and abnormal T cells. Mitochondrial dysfunction, energy metabolism and telomere alterations contribute at a molecular level to the increased oxidative stress in SLE.

Ox-LDLs stimulate endothelial activation, resulting in the increased expression of leukocyte adhesion molecules (VCAM-1), intercellular adhesion molecules (ICAM-1) and chemoattractant cytokines such as IL-6 and IL-8. High levels of OX-LDL and HDL with a proinflammatory phenotype and reduced LDL uptake capacity (piHDL) in SLE, early events of atherosclerosis, are found in lupus patients, in association with coronary or peripheral arterial disease and carotid plaque [144,145].

As precursor cells repairing the vascular endothelium, endothelial progenitor cells (EPCs) belong to the protective mechanism. However, they are reduced in number and have impaired function in SLE [146,147].

### 4.3. Role of Innate Immune System

#### 4.3.1. Immune Complexes, Dendritic Cells and Macrophages in Vascular Damage and Atherosclerosis in Systemic Lupus Erythematosus

The immunopathogenetic mechanisms of SLE are numerous and may occur simultaneously. The pathogenesis of systemic SLE has historically been attributed to the formation of immune complexes in the subsequent inflammatory cascade generated by cells of the innate immune response and the complement system. When immunocomplexes are deposited, they activate the complement system, recruit granulocytes and trigger phagocytosis by neutrophils and macrophages. The death of these cells releases large amounts of lytic enzymes responsible for tissue damage. in SLE, immunocomplexes are involved in the pathogenesis of nephritis, cerebral vasculitis, skin lesions and cytopenia.

Macrophages are the principal cells involved in the clearance of dead cells, cellular fragments and immune complexes. Growing evidence documents that the compromised macrophage function in the clearance of cellular debris and of immune complexes is involved in the pathogenesis of SLE.

Plasmacytoid dendritic cells, famous antigen-presenting cells (APC), respond to and also secrete IFN-I; in SLE, the presence of nuclear antigens overstimulates these cells. Excess IFN-I promotes accelerated atherosclerosis in different ways: direct vascular damage, reduced vascular repair, the reduced generation of progenitor endothelial cells, the increased production of proinflammatory cytokines and the reduced maturation of vascular smooth muscle cells. This events lead to plaque ulceration, rupture or thrombosis [148].

Aberrant macrophages contribute to the pathogenesis of SLE in various ways, some of which may also be implied in vascular damage. Classically, macrophages and neutrophils play an important role translating immune complex deposition into active disease through their release of cytokines and proteolytic enzymes. Not only does the hyperactivity of aberrant macrophages contribute to SLE, but their reduced normal functions can cause tissue/vascular damage (mediated by specific immunity) or even their accelerated apoptosis and impaired apoptotic clearance.

Firstly, a defect in the ability of macrophages to phagocytose apoptotic cells has been described in SLE [149]. This defect causes apoptotic cells to remain in the microenvironment long enough for membrane permeabilization to occur, allowing the escape of self-antigens, including nucleosomes, contained in the apoptotic cells themselves. This condition is known as ‘late apoptosis’.

What remains is an apoptotic cell that has not been phagocytosed and the released self-antigens, which are removed by other cell types that are only secondarily involved in this elimination process: dendritic cells (DC). Once mature, they are able to present phagocytosed self-antigens to T lymphocytes, thereby activating the adaptive immune response [150].

The apoptosis-induced modification of nuclear autoantigens such as proteins, DNA and RNA leads to their recognition as non-self molecules or as a danger signals, therefore activating the inflammasome pathway. Immune cells, including plasmacytoid dendritic cells and macrophages, respond to nucleic-acid-containing immune complexes by producing high levels of inflammatory cytokines such as type I IFN. Impaired apoptotic clearance and the involved receptors have been studied in systemic lupus erythematosus [151]. Together, these mechanisms create a vicious cycle of chronic inflammation and cell death in SLE.

Similarly, late apoptotic cells can launch proatherogenic inflammatory responses, and apoptotic cell engulfment plays an important role in the pathogenesis of atherosclerotic plaques as well [152].

In SLE, an increased rate of monocyte/macrophage apoptosis is observed. Increased monocyte/macrophage apoptosis contributes to the pathogenesis of autoantibody formation and organ damage through both increased apoptotic load and impaired clearance (of apoptotic material) [153].

Scavenger cell apoptosis, such as death induced by autoreactive cytotoxic T cells, can play a pathogenetic role in SLE and contribute to the severity of the disease.

The pathogenesis of SLE is related not only to B cell and T cell abnormalities, but also to dysregulation of the two major macrophage phenotypes, M1 and M2 [154].

The same biological factors that promote the onset of SLE also promote the polarization of M1 macrophages, and the M2 phenotype is more closely correlated with the repair and remodeling phases following flares, but has no beneficial effect on patient prognosis.

Macrophage polarization is a reversible process that plays a role in the development of autoimmune diseases such as RA and SLE. A number of studies have been carried out which demonstrate the excellent therapeutic potential of regulating the M1/M2 balance [155].

M2b can be detected in serum samples from SLE patients and is associated with the deposition of immune complexes in SLE [156].

Defective M2 macrophages exacerbate the development of SLE by secreting uncontrolled cytokines and promoting the abnormal deposition of immune complexes, such as M2-like macrophages lacking the expression of heme oxygenase-1, which are found in lupus nephritis, a complication of SLE [157]. M2 macrophages correlate with fibrosis in SLE, as in end-stage renal disease. The selective inhibition of M2b activity could reduce the proinflammatory effect of SLE and tissue damage [158].

Studies have analyzed the role of macrophages in models of inducible lupus nephritis, and the treatment of kidney disease mediated by nephrotoxic antibodies with a CSF-1R inhibitor, which depletes macrophages, was proven to reduce proteinuria [159].

Finally, there may be a relationship between IFN-I and macrophage polarization in SLE. Type 1 interferon is involved in the pathogenesis of SLE via the JAK/STAT pathway.

The JAK/STAT pathway is also an important signaling pathway that regulates the direction of macrophage polarization. However, the results of such studies on STAT-mediated macrophage polarization are particularly contradictory, perhaps due to the heterogeneity of the M2 population, which can be further divided into four subtypes [160].

#### 4.3.2. Role of Neutrophils and NETs in SLE and Atherosclerosis

Neutrophils are the first line of defense against infection and normally contribute to neoangiogenesis, coagulation and tissue repair. Neutrophils can respond to pathogens in three different ways: phagocytosis, degranulation or the formation of neutrophil extracellular traps (NETs).

First discovered in SLE in 1986, a subset of neutrophils, low-density granulocytes (LDGs), are more abundant in SLE patients than in the general population [161]. Patients with SLE and an increased number of circulating LDGs demonstrate heightened prevalence of vasculitis, arterial inflammation and coronary plaque [162]. These neutrophils differ from normal neutrophils in their enhanced pro-inflammatory properties, including cytokine and IFN production, increased NET formation and reduced phagocytic capacity.

LDGs secrete proinflammatory cytokines involved in disease pathogenesis and in atherosclerosis: TNF-α, IFN-γ and type I IFN [91,162]. In SLE, LDGs display spontaneous NET formation and the secretion of CXCL8, a chemokine that attracts and activates neutrophils [162].

NET formation is highly influenced by IFN-I, and NETs are endowed with pathogenic potential in SLE [163]. They are sources of oxidized nucleic acids, which can become additional autoantigens inducing the formation of more immune complexes (NET formation is also induced by the immune complexes already present) [164]. NETs damage vessel walls, and LDGs can also directly damage endothelial cells by inducing a programmed cell death cascade involving the NET product matrix metalloproteinase. Of note, in SLE, increased NET formation and decreased NET clearance are observed [165].

#### 4.3.3. The Inflammasome in SLE and Atherosclerosis

NLRP3 probably has a role in the pathogenesis of atherosclerosis, acting as a proinflammatory molecule that triggers the activation of caspase-1 and pro-IL-1β, pro-IL-18, and the initiation of pyroptosis [166].

In SLE, dsDNA and its autoantibodies can trigger the activation of the NLRP3 inflammasome [167].

Growing evidence suggests that the NLRP3 inflammasome plays a critical role in the pathogenesis of SLE, particularly in the context of the aberrant activation of the innate and adaptive immune system [168]. The aberrant activation of the NLRP3 inflammasome has been shown in several cell types in SLE patients, including macrophages [169] and podocytes [170]. The activation of NLRP3 in the macrophages of SLE patients leads to the neutrophil-mediated release of NETs and impairs their clearance, causing NET accumulation [171]. The NLRP3 inflammasome in T follicular helper cells (Tfh) has been shown to be involved in B cell activation, leading to the production of high-affinity antibodies, and is correlated with disease activity [172]. Furthermore, there is an interplay between IFN-I and NLRP3, which has also been documented in monocytes of SLE patients [173].

### 4.4. Role of the Acquired Immunity

#### 4.4.1. Role of T Lymphocytes in SLE and Atherosclerosis

T lymphocytes contribute to the pathogenesis of SLE by amplifying the autoimmune response. In SLE, there is an altered distribution of T lymphocyte subpopulations, decreased production of IL-2, an increase in the pro-inflammatory cytokines IL-6, IL-17 and BAFF/BLyS, and a decrease in regulatory T lymphocytes. As in RA, T helper lymphocytes play a particularly important role. In contrast to RA, a role for T helper follicles (TFH) is observed, which are normally involved in B lymphocyte differentiation at the level of secondary lymphoid tissues, stimulating functional B cells and inducing the apoptosis of dysfunctional or autoreactive B clones. The activation of Tfh cells is associated with disease activity and autoantibody production in SLE [174].

Th17 lymphocytes participate in inflammation by producing IL-17 and IL-22, thereby recruiting neutrophils and monocytes. Double-negative T lymphocytes (CD4-CD8) are also involved. IL-17 levels are elevated in the blood of patients with SLE [175].

The T regulatory lymphocyte (Treg) Foxp3+ physiologically switches off the excessive and persistent inflammatory response and facilitates tolerance to self by suppressing self-reactive T and B lymphocytes by inducing their apoptosis through FAS-L activation.

These lymphocytes are stimulated by IL-2 alpha and TGF-beta and are in inverse concentration to Th17. In particular, in the presence of IL-6, resident dendritic cells stimulate the differentiation of T lymphocytes naive to Tregs, whereas in an IL-6-rich microenvironment, differentiation towards Th17 is favored [176].

The imbalance in the Treg/Th17 ratio favors Th17. The ongoing inflammation increases the likelihood of endothelial damage and cardiovascular diseases.

#### 4.4.2. B Lymphocytes, Antibodies and CVD

The pathogenetic feature of SLE is the loss of self-tolerance with the formation of T and B autoreactive lymphocytes and the production of autoantibodies. The autoantibody targets include nuclear antigens, LPL, ox-LDL and anti-B2GPI.

ANA antibodies may play a role in the development of endothelial dysfunction and atherosclerosis. Some studies have found an inverse association between ANA titer (>160) and carotid compliance in women [177]. They are also associated with an increased rate of cardiovascular events and mortality [100].

Anti-dsDNA antibodies contribute to the pathogenesis of atherosclerosis, promoting a characteristic pattern or immune and vascular activation; they trigger cholesterol accumulation in macrophages and smooth muscle cells, are cytotoxic and induce inflammatory cytokine production [178].

In addition, anti-C1q antibodies can be found in patients with SLE, and these antibodies reduce C1q function and reduce the clearance of immune complexes; these antibodies are also considered to be predictive of lupus nephritis [179].

Lipoprotein lipase is an endothelial surface enzyme that hydrolyzes the triglycerides in VLDL and chylomicrons into free fatty acids and monoglycerides. Autoantibodies against lipoprotein lipase (LPL) have been found in 47% of patients with SLE [180].

The defect in lipoprotein metabolism contributes to the development of atherosclerotic lesions [181].

Atherothrombotic phenomena were first observed in patients with antiphospholipid syndrome, in whom local ROS production by inflammatory cells induces LDL oxidation. Ox-LDLs act as a chemotactic stimulus for monocytes and macrophages and they accumulate in foam cells. Ox-LDLs bind to Beta2GPI and form complexes against which autoantibodies are produced. These complexes, as well as Ox-LDLs alone, act as DAMPs and activate the inflammasome [182]. Inflammasome activation increases IL-1β production, which contributes to atherogenic inflammation.

The potential culprit of excessive B cell activation seems to play a role not only in SLE, but also in the development of atherosclerosis. Distinct B cell phenotypes may exert different functions in atherogenesis, with a B1 phenotype being atheroprotective and a B2 phenotype being pro-atherogenic [183].

Finally, B cell activation factor (BAFF) enhances autoreactive B lymphocyte proliferation and survival, leading to the production of a wide range of autoantibodies in SLE. Belimumab, which specifically targets BAFF, is the only B cell depletion therapy approved for SLE.

A genetic variant of BAFF causes increased production of BAFF and excess B cells and antibodies, and SLE carriers present high rates of arthritis, hematological alterations and nephritis, therefore increasing susceptibility to secondary CVD [143]. Also, B cell depletion therapy has been proven to reduce hypertension in experimental models of SLE [184].

### 4.5. Defects of DNA Repair and Immunosenescence

Cellular senescence phenomena are observed in SLE [185], which may also contribute to cardiovascular damage. The defect in DNA repair is particularly evident, as ultraviolet radiation is one of the most notorious of the environmental stimuli that can induce disease flares. In most patients, sun exposure induces keratinocyte ptosis and alters DNA and several cellular proteins, making them antigenic.

The DNA repair defect characteristic of senescence [186] is perhaps the greatest risk factor for the development of oxidative damage in vascular endothelial cells. This defect is exemplified by ataxia-telangiectasia, an autosomal recessive cerebellar ataxia or genomic instability syndrome. In this disease, pro-inflammatory substances such as ox-LDL and TNF-α induce telomere inactivation [187].

At the vascular level, these patients show endothelial dysfunction, accelerated atherosclerosis [188], vascular dysplasia with loss of elastic fibers and the proliferation of smooth muscle cells, leading to telangiectasia and early coronary artery disease [189]. In addition, the impaired DNA damage repair system in SLE activates the innate immune response; unrepaired damaged DNA accumulates in the cytosol, activating PAMPs that stimulate the production of IFN, cytokines and chemokines [190]. The altered DNA contains mutations that can act as neoantigens and stimulate antibody production [191].

Lastly, anti-dsDNA antibodies increase cardiovascular risk in SLE by defining a characteristic pattern of immune and vascular activation that includes altered monocytes with a tendency toward apoptosis, pro-inflammatory neutrophils with an increased tendency to form NETs, activated endothelial cells incapable of adequate NO production and the excessive production of pro-thrombotic and pro-inflammatory molecules that also stimulate pro-atherogenic dyslipidemia [192].

### 4.6. Kidney Damage and Atherosclerosis

Chronic kidney disease (CKD) is an independent risk factor for early mortality from cardiovascular causes. Even in the early stages of the disease, there is a risk of accelerated atherosclerosis [193].

Statins, which do not guarantee a reduction in the risk of atherosclerosis in the later stages of the disease, prove beneficial in the early stages of chronic kidney disease in the general population with CKD.

Factors that may contribute to atherosclerosis in CKD are [194] endothelial dysfunction, chronic inflammation, the pro-inflammatory cytokine pool and ROS, largely produced by activated macrophages, immunosenescence and abnormalities of the lipid profile, such as elevated LDL and lipoprotein A (Lp(a)) levels, typical of nephrotic syndrome. When kidney damage does not lead to the development of nephrotic syndrome and the lipid profile is not quantitatively altered [195], the ox-LDLs are still significantly increased (in CKD) [196].

Renal involvement is very common in patients with SLE, affecting 40–50% of patients. It is often the most severe manifestation of SLE. Asymptomatic in the majority of cases, it can lead to proliferative forms of glomerular damage with proteinuria and hematuria, often as part of a nephrotic syndrome in which secondary hypertension and dyslipidemia develop, both of which predispose patients to accelerated atherosclerosis. In fact, lupus nephritis is associated with a 9-fold increased risk of atherosclerotic cardiovascular disease compared to the general population [197].

Patients with lupus nephritis may be twice as likely to develop atherosclerotic plaques as patients with lupus without nephropathy [198]. Severe renal atherosclerosis is also common in patients with IgA nephropathy; note that most patients are female, a population classically protected from cardiovascular damage [199].

### 4.7. Libman–Sacks Endocarditis and Vascular Damage

Libman–Sacks syndrome is a benign form of endocarditis associated with SLE and with APS [200]. Histologically, the lesions of Libman-Sacks endocarditis are small sub-centimeter fibrinous vegetations, with varying degrees of neovascularization, inflammatory infiltrate of mononuclear cells, fibrous plaque formation, scarring and focal calcifications [201]. The exact pathogenesis of these lesions is unclear, but it may have similar mechanisms to the endothelial dysfunction observed in the blood vessels of patients with APS, and, in particular, may be related to the local deposition of anti-beta2-GPI antibody complexes with the beta2-GP1 receptors of heart valve endothelial cells [202].

### 4.8. Antiphospholipid Syndrome and Atherosclerosis

Antiphospholipid antibody syndrome (APS) is characterized by venous or arterial thromboembolism, poly-embolism and hemorrhagic risk in the presence of the antiphospholipid antibodies (aPL) lupus anticoagulant (LA), anti-cardiolipin antibodies (aCL) or anti-beta2glycoprotein (B2GPI). Its diagnosis requires the simultaneous presence of at least one clinical and one laboratory criterion [203].

Antiphospholipid antibodies are not specific to SLE, but their presence is a diagnostic criterion for SLE. The prevalence of major clinical features in cohort studies of SLE patients with APS is as follows: 13% stroke, 11% myocardial infarction, 9.5% deep vein thrombosis and 6% pregnancy complications [204].

Physiologically, endothelial damage activates coagulation in addition to the inflammatory response. In APS, endothelial dysfunction is a key pathological component responsible for accelerated atherosclerosis [205], deep vein thrombosis, pulmonary embolism, pulmonary arterial hypertension, proliferative nephropathy, cerebral vascular occlusion, endocarditis and cardiac valvopathy, pre-eclampsia and placental insufficiency.

Among the various pathogenetic mechanisms proposed for APS, in addition to specifically procoagulant mechanisms, such as the inhibition of anticoagulant protein C and the increased platelet production of thromboxane (TXA2), at the endothelial level, increased platelet aggregation and increased proinflammatory activity have been identified [206].

The increased expression of adhesion molecules leads to decreased function of nitric oxide synthase (eNOS) with reduced NO production. aPL antagonizes the phosphorylation of the eNOS enzyme and also reduces the production of other vasodilators derived from arachidonic acid, including eicosanoids such as prostacyclin and thromboxane (TXA2).

In APS, interactions between the endothelium and monocytes are increased [207]; aPL can directly activate monocytes and produce chemoattractant substances (MCP-1) [208].

Monocytes adopt a pro-inflammatory phenotype with high energy consumption and oxidative stress. APS triggers complement activation and subsequent chemotaxis [209], which generates high local oxidative stress with the arrival of granulocytes and macrophages.

In addition to accelerated peripheral and coronary atherosclerosis [210], APS is associated with increased endothelial cell proliferation, intimal hyperplasia and non-atherosclerotic vascular stenosis compared to the general population with comparable risk factors. Endothelial hyperplasia has also been shown in renal biopsies from patients with APS and mTOR activation. Such activation is associated with severe vasoconstriction in catastrophic APS syndrome [211].

The role of endothelial function assessment for risk stratification and potential therapeutic implications in APS needs to be explored in further studies [212]. In addition to antiplatelet, anticoagulant [213] and anti-malarial drugs, statins have been proposed for their immunomodulatory and anti-inflammatory effects in APS syndrome, particularly in patients with an altered lipid profile with APS who are refractory to anticoagulation [214]. Vascular dysfunction plays a cardinal role in the pathogenesis of APS, and further studies are needed in order to stratify the risk of these patients and to evaluate possible therapies targeted toward the endothelium.

### 4.9. Brief Notes on Therapies for Systemic Lupus Erythematosus and Their Cardiovascular Effects

Compared with other rheumatic diseases, there has been very little therapeutic innovation in SLE. SLE therapies also have cardiovascular effects, and drug therapy in SLE is a double-edged sword. Long-term glucocorticoid therapy is associated with a high risk of atherosclerosis, dyslipidemia, diabetes, heart failure and myocardial infarction [174]. According to the EULAR recommendations, corticosteroids should be reduced to a maintenance dose of ≤5 mg/day (prednisone equivalent) and as soon as possible [215]. A steroid-sparing approach is recommended, with immunosuppressants. However, side effects and toxicity limit the use of these drugs.

Like steroids, azathioprine [216] and cyclophosphamide [217] carry proatherogenic risk, as they can aggravate endothelial dysfunction and plaque formation; calcineurin inhibitors are known to cause hypertension and endothelial dysfunction. [218]. On the other hand, both hydroxychloroquine and mycophenolate [219] have a cardiovascular protective effect [220]. Hydroxychloroquine reduces IFN-I production and improves the lipid profile, reducing LDL and insulin resistance [221]. In the case of Mycophenolate, not all trials have shown a CV benefit [222].

Hydroxychloroquine, an antimalarial drug, is the oldest disease-modifying anti-rheumatic drug and has been part of SLE therapy and lupus nephritis for decades [223]. Its complex mechanism of action includes interference with phagocytosis, antibody production and the selective presentation of self-antigens, and bro blocks monocyte and T lymphocyte proliferation and reduces the production of the cytokines TNF-α, IL-6, IL-17, IFN-alpha and gamma [221]. Hydroxychloroquine reduces the activity of nicotinamide adenine dinucleotide phosphate oxidase (NADPH), which generates ROS, so hydroxychloroquine reduces endothelial dysfunction [224]. Given its beneficial effect on SLE and prevention of cardiovascular damage, EULAR has included hydroxychloroquine as a recommended treatment in the management of SLE [225].

Biologic drugs should be considered as first-line therapy in patients not responding to hydroxychloroquine (alone or in combination with glucocorticoids) or patients unable to reduce glucocorticoids, but the specific cardiovascular effects of these medications need to be further explored [226].

## 5. Conclusions

Despite increasingly effective therapies, cardiovascular risk is increased in patients with rheumatic diseases, particularly SLE and RA, compared with the general population.

Traditional cardiovascular risk assessment scores fail to estimate the CVD risk associated with SLE and RA. These diseases are independent CV risk factors. Accelerated atherosclerosis is caused by an interplay of traditional and disease-specific risk factors.

The EULAR guidelines propose the use of a multiplication factor of 1.5 to estimate the cardiovascular risk in RA patients with a disease duration of more than 10 years, with extraarticular manifestations and positive serology for RF or ACPA.

SLE, lupus nephritis and APS convey an increased risk compared to RA, but international scientific societies have not endorsed the use of a specific CVD risk assessment tool due to limitations and discrepancies in the evidence.

Clinicians need to integrate cardiovascular-risk-preventive strategies into their daily practice not only to focus on disease-specific interventions, but also for the promotion of smoking cessation, healthy lifestyles and the normalization of BMI.

The need for precise CV risk assessment tools is urgent.

Cohort clinical trials are required to investigate the cost-effectiveness of screening practices for subclinical atherosclerosis, dyslipidemia and the risk for heart failure. These trials may employ non-radiation diagnostic exams, carotid ultrasound and electrocardiogram pattern analysis to identify predictive signs for heart failure. The effect of lower cut-offs and a more aggressive treatment for cardiovascular comorbidities, such as hypertension and dyslipidemia, may be investigated.

Finally, novel cellular or molecular biomarkers predictive of cardiovascular disease risk can be identified through preclinical studies focused on the sophisticated immunological network.
